# TPX2 promotes gastric cancer progression and angiogenesis via Wnt/β-catenin signaling

**DOI:** 10.3389/fonc.2026.1764583

**Published:** 2026-02-13

**Authors:** Li Xu, Weibo Zhang

**Affiliations:** 1 Department of Oncology, The First People’ s Hospital of Tongxiang, Jiaxing, Zhejiang, China; 2 Department of Gastrointestinal and Anorectal Surgery, The First People’s Hospital of Tongxiang, Jiaxing, Zhejiang, China

**Keywords:** gastric cancer, targeting protein for Xklp2, tumor, tumor vessel formation, Wnt/β-catenin pathway

## Abstract

**Background:**

Among gastric cancer (GC) cases, stomach adenocarcinoma (STAD) comprises the majority, exceeding 95%. GC develops stealthily and quickly; the majority of cases are detected at advanced or mid-stage, which adversely affects the prognosis. Hence, delving into the molecular mechanisms contributing to GC advancement, and discovering innovative therapeutic targets and latent biomarkers for GC remain at the forefront of research.

**Methodology:**

QRT-PCR and western blot procedures were followed to make clear the targeting protein for Xklp2 (TPX2) expression in GC and surrounding non-tumor tissues. The impacts of TPX2 overproduction or attenuation on the biological traits of GC cells were examined through CCK-8 assay and transwell assay; meanwhile, vessel formation assays were employed to assess TPX2’s impact on the vascular formation of GC. Furthermore, TCF/LEF activity was determined using the Top-Flash reporter plasmid; the production of proteins linked to epithelial-mesenchymal transition (EMT) and proteins linked to the Wnt/β-catenin pathway was evaluated through western blot. A nude mouse xenograft tumor model was established, and pathological staining was performed to detect tumor proliferation and angiogenesis.

**Results:**

TPX2 level was much higher in GC tissues and cells. When overexpressed, TPX2 contributed to these cells’ viability, motility, infiltration, EMT, and angiogenesis, alongside activation of Wnt/β-catenin pathway; whereas, when silenced, TPX2 caused the opposite outcomes. Additionally, TPX2 overproduction enhanced the TCF/LEF transcriptional activity, while the introduction of a Wnt/β-catenin pathway inhibitor partly mitigated the GC advancement enhanced by overproducing TPX2. This phenomenon further confirms that TPX2 exerts carcinogenesis by enabling the Wnt/β-catenin pathway. Silencing TPX2 reduced tumor volume and weight in nude mice, inhibited angiogenesis and proliferation, and suppressed EMT *in vivo*.

**Conclusion:**

By activating the Wnt/β-catenin pathway, TPX2 promotes GC migration, invasion, and tumor angiogenesis, which collectively drive GC malignant progression.

## Highlights

- TPX2 level is substantially heightened in GC.
- TPX2 overexpression contributes to GC cells’ growth, motility, infiltration, EMT, and angiogenesis, alongside activation of Wnt/β-catenin pathway.
- Silencing TPX2 hampers the malignant activities of GC and blocks Wnt/β-catenin pathway.
- Introducing a Wnt/β-catenin pathway inhibitor partly mitigates the GC aggravation enhanced by TPX2 overproduction.

## 1 Introduction

The globally fourth most frequent malignant tumor is gastric cancer (GC), which is also the third major contributor to global death from cancers, causing approximately 800,000 deaths annually (1–3). Over 95% of GC cases, when classified according to anatomical positions and histological characteristics, fall into the category of stomach adenocarcinomas (STAD) (4). Radical operation remains the fundamental GC treatment method, yet more than half of the patients face relapses within five years after surgery, resulting in unfavorable outcomes (5, 6). Additionally, owing to the covert nature and swift advancement of GC, the majority of patients are diagnosed in later stages, missing the prime window for surgical intervention (7, 8). Therefore, intensifying research into the molecular underpinnings of GC development and prioritizing early diagnosis and interventions are pivotal in diminishing the rates of recurrence and mortality.

Targeting protein for Xklp2 (TPX2), a protein associated with microtubules, is essential for mitosis and spindle assembly (9–11). Controlled by cell cycles, TPX2 is produced in the G1-S stage of a cell cycle and vanishes post-mitosis. Structural damage to TPX2 may cause improper spindle assembly, which in turn triggers chromosomal abnormalities, inducing tumors (12–14). Increasing evidence suggests a connection between aberrant TPX2 production and cancer aggravation, including cancers at the pancreas (15), prostate (16), bladder (17), and esophagus (18); identifying TPX2 as a diagnostic and prognostic indicator for malignancies. Besides, a study uncovered that TPX2 when overproduced, enhances the exacerbation of GC and lowers the survival rates (19). However, inadequate investigations are made into the precise mechanisms of how TPX2 mediates the worsening of GC.

The Wnt/β-catenin pathway, as an essential regulator of embryonic development and cellular homeostasis, plays a vital role in the advancement of various digestive system tumors through its abnormal activation (20, 21). Earlier studies have revealed that sustained stimulation of this pathway causes the aggregation of β-catenin within the cell nucleus, thereby regulating target genes levels including c-Myc and Cyclin D1, encouraging epithelial-mesenchymal transition (EMT) (22, 23). Moreover, the activated Wnt/β-catenin pathway can accelerate tumor progression and distant metastasis by promoting angiogenesis in GC (24). Studies have indicated that TPX2 overexpression can stimulate the Wnt/β-catenin pathway, diminish the chemotherapy sensitivity of lung cancer cells, and accelerate tumor cell metastasis (25). It remains unclear whether TPX2 regulates the malignant progression of STAD via the Wnt/β-catenin pathway.

This study initially determined the TPX2 production in GC patient tissues and different cell types and then delved into the interference of TPX2 overproduction or silencing on GC’s biological traits and vascular formation. At last, a pathway inhibitor was employed to further investigate whether TPX2 governs GC worsening via the Wnt/β-catenin pathway or not. This investigation seeks to define the specific roles TPX2 plays in the exacerbation of GC, thereby inspiring new ideas for its early diagnosis and targeted treatment.

## 2 Methodology and materials

### 2.1 Clinical tissue samples

We collected cancerous and paracancerous normal tissue samples from 42 STAD patients admitted to The First People’ s Hospital of Tongxiang, of which 8 patients were from stage I, 12 patients from stage II, 15 patients from stage III, and 7 patients from stage IV. Parancerous tissues are adjacent normal gastric mucosal tissues ≥2 cm from the tumor margins, with no infiltration of cancer cells, obvious inflammation or heterogeneous hyperplasia as confirmed by pathology. These patients did not undergo radiotherapy before surgery, and the specimens after surgical resection were divided and labeled within 30 min, immediately put into liquid nitrogen quick-freezing, and subsequently transferred to -80°C refrigerator for long-term storage. The investigation received approval from the Ethics Committee of the First People’ s Hospital of Tongxiang (No. T202509116), and informed consent for sample acquisition was garnered from all the participating patients as prescribed.

### 2.2 Cell culture and transfection

Human umbilical vein endothelial cells (HUVECs, CP-H082), normal gastric epithelial cells (GES-1, CL-0563), and GC cell lines HGC-27 (CL-0107), MKN-45 (CL-0292), and AGS (CL-0022) were obtained from Wuhan Procell System (Hubei, China). The cells were maintained in a culture environment of high-glucose DMEM (containing antibiotics, PM150210A, Wuhan Procell System), with 10% fetal bovine serum (FBS, 164210, Wuhan Procell System), setting to 37 °C and 5% CO_2_. The medium was exchanged on alternate days and cells were passaged every three days. TPX2 overexpression plasmids (OE-TPX2), TPX2 short hairpin RNA (Sh-TPX2), and negative controls (OE-NC and Sh-NC) were prepared by Sangon Biotech (Shanghai, China). According to the Lipofectamine 3000 (L3000001, Invitrogen, Carlsbad, CA, USA) instructions, 2 μg of plasmid DNA was mixed with 4 μL of P3000 reagent in 125 μL of Opti-MEM medium (51985091, Invitrogen). Another 4 μL of Lipofectamine 3000 was taken and diluted with 125 μL of Opti-MEM medium. The two mixtures was gently mixed and incubated at room temperature for 15 min to form a stable DNA-Lipofectamine 3000 complex, which was subsequently transfected into AGS and MKN-45 cells and cultured for 48 h.

### 2.3 Quantitative RT-PCR assays

The extraction of total RNA from GC tissues and cells was initially performed using the Trizol reagent (15596018CN, Invitrogen), and cDNA was subsequently generated through reverse transcription with AMV reverse transcriptase (B600020, Sangon Biotech). PCR amplification of the cDNA was then conducted using TB Green FAST qPCR Kit (CN830S, TAKARA, Tokyo, Japan). The relative expression of the target gene TPX2 was calculated by data normalization analysis using GAPDH as an internal reference gene and the 2 ^-ΔΔCt^ method. Biological replicates were 42 (cancerous tissues and corresponding paracancerous tissues from 42 patients), 3 technical replicates were performed and the mean value was taken as the final data for statistical analysis. Below are the detailed primer sequences:

GAPDH: F: 5’-GGAGCGAGATCCCTCCAAAAT-3’; R: 5’-GGCTGTTGTCATACTTCTCATGG-3’.
TPX2: F: 5’-ATGGAACTGGAGGGCTTTTTC-3’; R: 5’-TGTTGTCAACTGGTTTCAAAGGT-3’.

### 2.4 CCK-8 assays

AGS and MKN-45 cells in logarithmic growth phase were taken and inoculated in 96-well cell culture plates at a density of 1.5×10^4^ cells/well. Cell proliferation capacity at different time points was detected using independent culture plates. 96-well plates were set up for four time points: 0 d, 1 d, 2 d, and 3 d. The plates at each time point were seeded and cultured simultaneously to avoid interference with cell viability from multiple detections in the same well. After the cells were attached to the wall (about 24 h), the old medium was discarded and replaced with fresh complete medium containing 10% CCK-8 reagent (C917226, Macklin, Shanghai, China) and incubated for 2 h at 37 °C (26). Ultimately, a microplate reader was used to read the OD_450_ values of the cells.

### 2.5 Transwell assays

First, a 1:6 dilution of Matrigel (B718495, Macklin) with serum-free DMEM was prepared, and then 100 µL of this mixture was deposited into each transwell chamber (8 μm, Corning, Tewksbury, MA, USA), followed by one-night incubation in a culture incubator. The next day, any fluid remaining in the units was aspirated, and serum-free DMEM was introduced to hydrate the basement membrane. Then, the cell suspension (200 µL, 2×10^5^ cells/mL) was placed into each transwell unit, with the space below it receiving DMEM with 10% FBS. After 36-hour incubation, matrix gel and cells in the unit were removed; cells in the lower space were exposed to 4% paraformaldehyde (P885233, Macklin) for half an hour, and treated with 0.1% crystal violet (C916088, Macklin) for 5 min, followed by two PBS washes. Finally, five non-overlapping fields of view were randomly selected from each transwell chamber for photographing using a CX33 Optical Microscope (Olympus, Tokyo, Japan). The number of cells in each field of view was counted, and cells with blurred edges and severely overlapping cell clusters were excluded. The average number of cells in the five fields of view was taken as the final number of invasive cells.

The migration assay followed the same steps as the mentioned invasion assay, with the only difference being the non-addition of matrigel to the transwell unit.

### 2.6 Top-flash assays on the TCF/LEF activity

The β-catenin dependent TCF/LEF transcriptional activities were assessed using the TOP-Flash reporter gene system, with the TOP-Flash vector (D2501) sourced from Beyotime (Shanghai, China). In detail, MKN-45 and AGS cells were co-transfected with the TOP-Flash vector, OE-TPX2, Sh-TPX2, OE-NC, and Sh-NC separately using Lipofectamine 3000. 48 h after transfection, the cells were washed twice with PBS, cell lysis buffer was added, and the cells were lysed on ice for 30 min. The cells were then centrifuged and the supernatant (containing luciferase protein) was collected. Luciferase activity was then detected using the Dual-Lucy Assay Kit (RG027, Beyotime): 20 μL of supernatant was added to a 96-well plate, followed by 100 μL of firefly luciferase substrate. After mixing, the firefly luciferase activity (FL) was immediately detected. Subsequently, 100 μL of Renilla luciferase substrate was added, mixed, and the Renilla luciferase activity (RL) was detected. FL/RL was used as the final quantification of TCF/LEF transcriptional activity.

### 2.7 Vessel formation assays

AGS and MKN-45 cells were seeded in 12-well plates (2×10^5^ cells/well). When the cell confluence reached 80%, the original culture medium was discarded, and the cells were washed twice with serum-free DMEM medium. Then, serum-free DMEM medium was added and the cells were cultured for another 24 h. At the end of culture, the supernatant of each well was collected and centrifuged to remove cell debris and impurities. The supernatant was taken and filtered through 0.22 μm sterile filter membrane to remove bacteria and obtain GC cell conditioned medium. A 96-well plate was filled with 50 μL per well of liquid matrigel, then underwent a 1-hour incubation at 37 °C. Meanwhile, logarithmic growth stage HUVECs were taken, and the cells were resuspended with prepared STAD cell conditioned medium, adjusted the cell concentration to 5×10^3^ cells/well, and placed in the incubator for 24 h co-incubation with conditioned medium. HUVECs cell suspension pretreated with conditioned medium for 24 h was added to a 96-well plate coated with Matrigel, gently shaken to mix, and incubated at 37 °C for 8 h. Finally, microscopic observation of vessel formation was conducted. Quantitative analysis was performed using ImageJ software (National Institute of Mental Health, Bethesda, MD, USA). The cumulative length (in μm) of all tubular structures in each field of view was measured, and the average of the cumulative lengths of the five fields of view was taken as the final quantitative result of the sample.

### 2.8 ELISA

Cells AGS and MKN-45 were grown in 6-well plates and after 48 h, centrifuged and the supernatant was treated per the instructions of the Vascular Endothelial Growth Factor (VEGF) ELISA kit (PV963, Beyotime). Afterward, the absorbance of VEGF in this supernatant was read by a microplate reader. Whereafter, a standard curve was plotted and the concentration of VEGF was worked out.

### 2.9 Molecular docking

The 3D structure files of TPX2 and β-catenin protein were acquired via the PDB database (https://www.rcsb.org/). The structure of both proteins were separately processed using PyMOL 2.5.0 (Schrödinger, Inc., New York, USA) and AutoDock Tools 1.5.7 (Scripps Research, California, USA), respectively. Subsequently, the ZDOCK platform (https://zdock.wenglab.org/) was used for rigid molecular docking simulations, and the Z-score value was used as the core screening criterion to screen the optimal complex model for the combination of the two.

### 2.10 Nude mouse tumor model

BALB/c nude mice (12~15 g, 4~5 weeks old) were purchased from Vitalriver (Beijing, China) and maintained at 22 °C with 45~50% humidity under a 12-hour light/dark cycle. After acclimating the nude mice for one week, they were divided into Sh-NC and Sh-TPX2 groups (n=5) using the random number table method. A 100 μL injection of Sh-NC- or Sh-TPX2-transfected AGS cells (2×10^7^ cells/mL) was administered into the right axillary region of mouse. Weekly tumor growth monitoring involves measuring and documenting tumor size via a vernier caliper to construct a tumor growth curve. After 28 d, mice were anesthetized and euthanized through cervical dislocation. Tumor tissue was excised, weighed, and photographed for documentation. The experiment employed a single-blind method: one researcher performed cell seeding, while another researcher, unaware of the group assignments, was responsible for subsequent tumor growth monitoring, size measurement, data recording, and tumor tissue processing. To ensure animal welfare, the following humane endpoints were set, and mice were euthanized immediately if any of the following conditions were observed: 1. Tumor diameter ≥ 1.5 cm or volume ≥ 1000 mm^3^; 2. Weight loss exceeding 20% from pre-inoculation levels, with no recovery for 3 consecutive days; 3. Significant signs of cachexia, including lethargy, limited activity, and inability to eat or drink normally; 4. Tumor ulceration, bleeding, infection, or metastasis to other sites. If any of the following situations occur during the experiment, the mouse data will be excluded and a new experimental animal will be added (this situation did not occur in this experiment): 1. No tumor formation within 7 days after inoculation; 2. Severe infection, redness, swelling and ulceration at the inoculation site, affecting tumor growth monitoring; 3. The mouse dies unexpectedly; 4. Tumor volume measurement data is missing or abnormal. The animal experimental protocol of this study has been approved by The First People’ s Hospital of Tongxiang Ethics Committee (No. TX202506113).

### 2.11 Hematoxylin and eosin staining

The tumor tissue from nude mice was immersed in 4% paraformaldehyde for 24 hours for fixation. It was then dehydrated using a gradient of ethanol solutions at 100%, 95%, 75%, and 50% concentrations. The tumor tissue was embedded in paraffin and sectioned into 4 μm thick serial sections. Subsequently, sections were dewaxed in xylene (X821391, Macklin), hydrated with a gradient of ethanol solutions, and then immersed in hematoxylin stain (C0105S, Beyotime) for 8 minutes. Excess stain was rinsed off with tap water, followed by differentiation with differentiation solution (C0161S, Beyotime) for 20 seconds. The sections were then gently washed with tap water immediately, and immersed in eosin staining solution (C0106S, Beyotime) for 60 s. After staining, the tissue sections were placed in 70% ethanol for dehydration and examined using a microscope.

### 2.12 Immunohistochemistry

Paraffin-embedded tumor tissue sections were dewaxed, hydrated with graded ethanol solutions, and placed in a citrate buffer solution (0.1 mol/L, pH=6) for microwave-assisted antigen retrieval. The sections were incubated in a 3% H_2_O_2_ solution for 30 min, then they underwent thorough washing with PBS. The sections were uniformly coated with 5% bovine serum albumin (BSA, A801320, Macklin) and blocked for half an hour, and then incubated primary antibody Ki67 (PA5-19462, 1:100, Invitrogen), CD31 (MA5-37858, 1:100, Invitrogen) or VEGF (MA5-32038, 1:50, Invitrogen) at 37 °C for 90 min. Subsequently, sections were treated with goat anti-rabbit IgG (31460, 1:1000, Invitrogen) for 1.5 h in a dark environment at 25 °C. DAB solution (P0203, Beyotime) was applied for the color development reaction. When the desired color intensity was achieved, the reaction was immediately stopped by adding distilled water. The samples were re-stained with hematoxylin solution, mounted with Neutral Balsam (C0173, Beyotime), air-dried, and then observed under a microscope.

### 2.13 Western blot assays

The RIPA lysis buffer (R0020, Solarbio) was added to GC tissues and cells to lyse and extract proteins, which were then measured by a BCA protein assay kit (B917925, Macklin) (27). After being treated by gel electrophoresis, the proteins were transferred onto a PVDF membrane (Invitrogen) and blocked. Two hour later, it underwent an overnight incubation with primary antibodies against TPX2 (ab264124, 1:10000, Abcam, Cambridge, MA, USA), Snail (MA5-14801, 1:1000, Invitrogen), E-cadherin (ab40772, 1:1000, Abcam), Slug (PA5-20289, 1:1000, Invitrogen), β-catenin (13-8400, 1:500, Invitrogen), VEGF (MA5-32038, 1:500, Invitrogen), N-cadherin (ab18203, 1:1000, Abcam), and Hypoxia-inducible factor-1α (HIF-1α) (PA1-16601, 1:100, Invitrogen) at 4 °C. The membranes were then treated with goat anti-rabbit IgG (31460, 1:10000, Invitrogen) for 90 min, then developed and exposed. Then, the obtained protein bands were processed using Image J software, obtaining the grayscales, with GAPDH (ab181603, 1:10000, Abcam) as the internal control.

### 2.14 Statistical analysis

All assays were performed with three repetitions at least, with outcomes presented as mean ± standard deviation. Prism software (Graphpad 9.0) and Figdraw (https://www.figdraw.com/static/index.html#/) was employed for plotting. Statistical analyses were conducted through SPSS 26.0 software (IBM SPSS Statistics 26). Comparison between two groups: normality and homogeneity of variance tests were performed first; if the data conformed to normal distribution and have homogeneity of variance, independent samples t-test was used; if it did not, nonparametric Mann-Whitney U test was used. Comparisons among multiple groups: One-way ANOVA was used. When comparing pairs of groups, the LSD test was used for groups with homogeneous variances and the Tamhane test was used for groups with unequal variances. Tumor growth curves: Repeated measures ANOVA was used, and differences between groups were analyzed by Bonferroni *post-hoc* test. *P<0.05 signifies a notable difference.

## 3 Results

### 3.1 TPX2 production in GC

**Figure 1:**
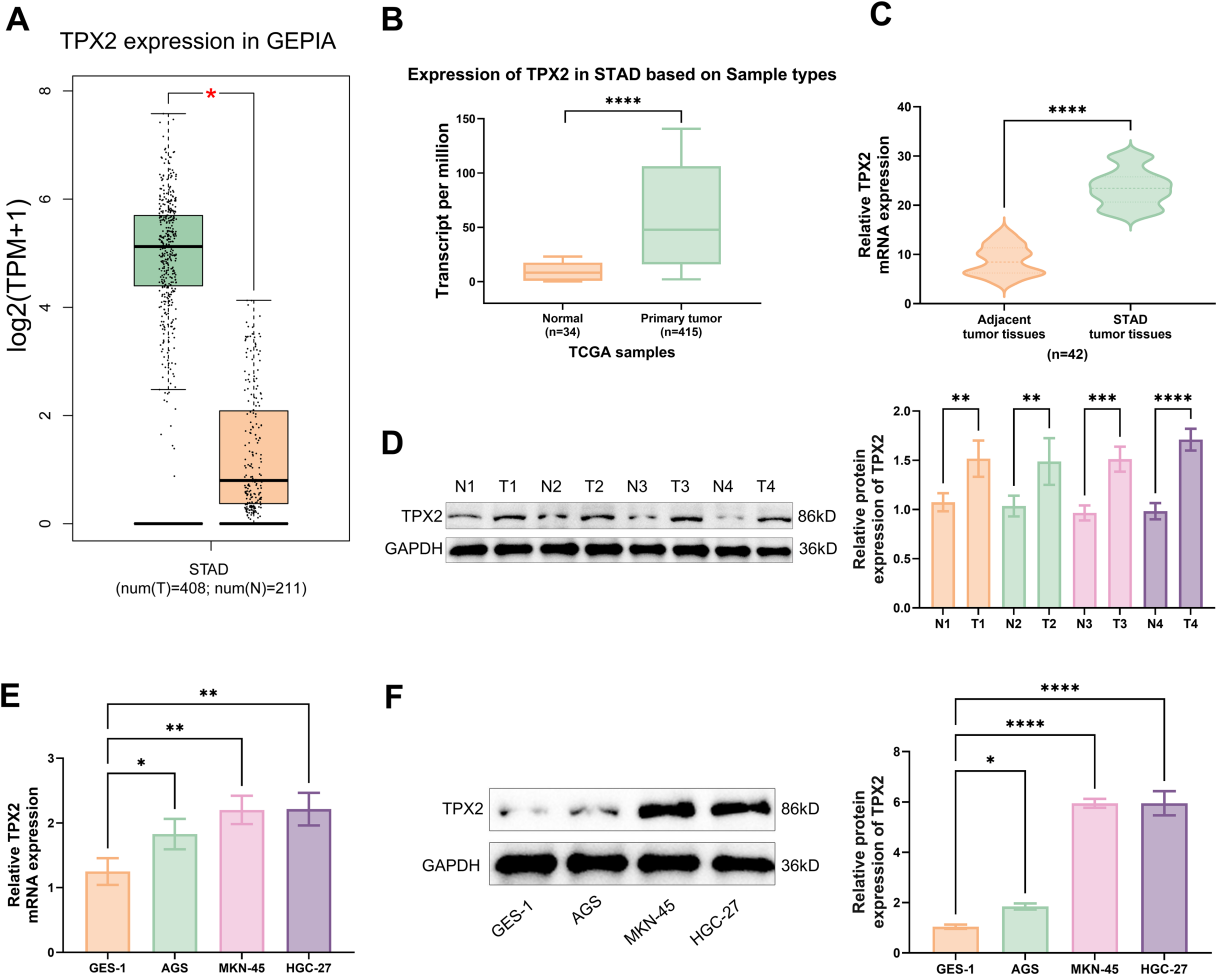
TPX2 production in GC. **(A)** TPX2 production within the GEPIA database. **(B)** TPX2 production within the TCGA database. **(C)** qRT-PCR was utilized for detecting TPX2 level in GC and surrounding normal tissues. **(D)** Detection of TPX2 protein levels in GC and surrounding normal tissues by western blot. **(E)** Outcomes from qRT-PCR assays on the TPX2 production in different cells. **(F)** Outcomes from western blot assays on the TPX2 protein production in different cells. *p<0.05, **p<0.01, ***p<0.001, ****p<0.0001.

TPX2 production is substantially heightened in STAD, as recorded in databases GEPIA (http://gepia.cancer-pku.cn/) and TCGA (https://www.cancer.gov/ccg/research/genome-sequencing/tcga) (**Figures 1A, B**). According to the TPX2 production in different tissues tested by qRT-PCR, STAD tissues displayed an enhancement in TPX2 production while the surrounding normal tissues showed a low TPX2 production level (**Figure 1C**). Beyond that, four randomly selected samples from tumor tissues (T) and surrounding normal tissues (N) of STAD patients were examined through western blotting to understand the TPX2 protein production. Consequently, STAD tissues demonstrated a TPX2 protein production greatly superior to normal tissues (**Figure 1D**). Besides, TPX2 mRNA and protein were found to be remarkably raised in GC cell lines (MKN-45, AGS, and HGC-27) (**Figures 1E, F**). Thus, subsequent assays were performed using cells AGS and MKN-45.

### 3.2 The interference of TPX2 overproduction with the biological activities of GC cells

**Figure 2:**
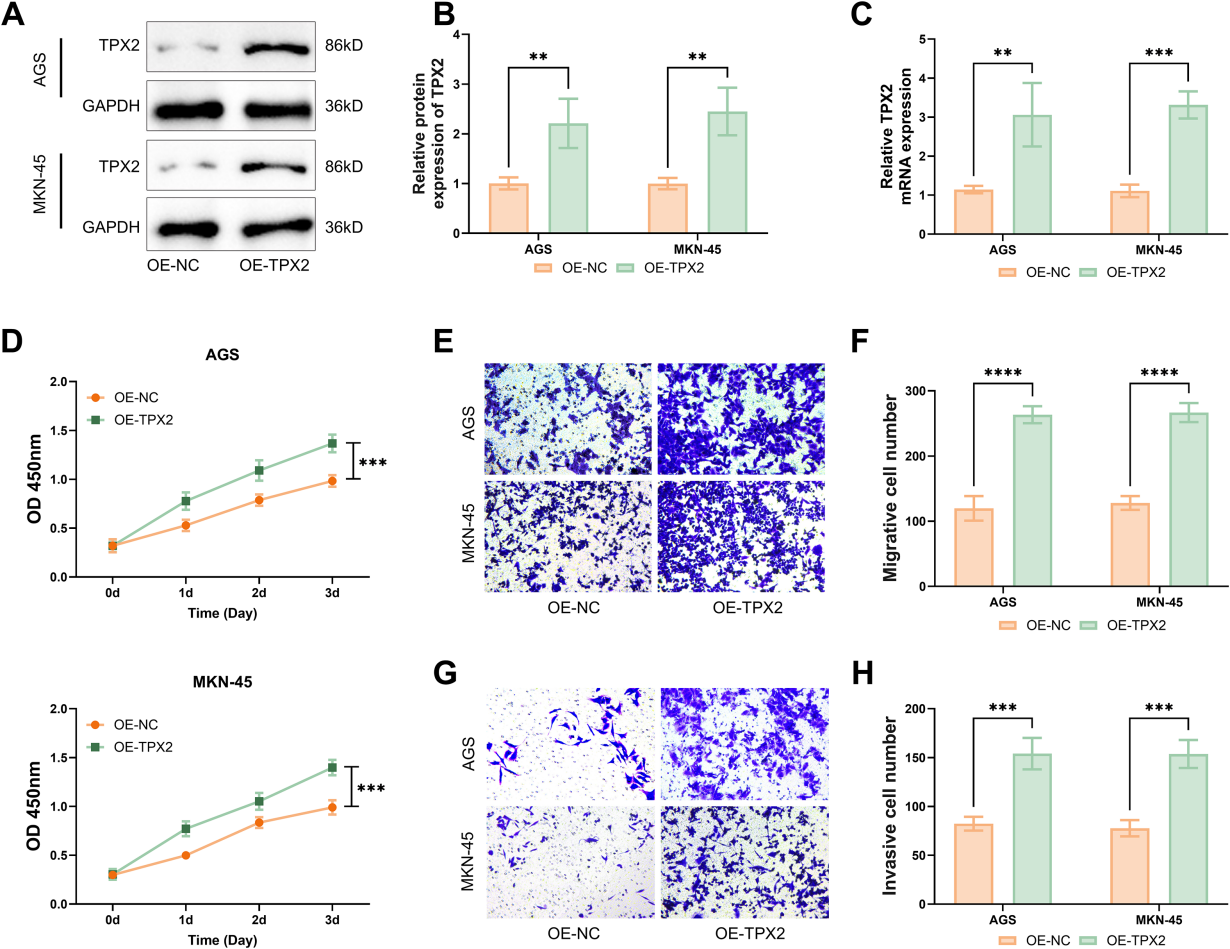
The interference of TPX2 overproduction with the biological activities of GC cells. **(A, B)** OE-TPX2 was transfected into AGS and MKN-45 cells, and TPX2 overexpression efficiency was assessed through western blot. **(C)** Outcomes from qRT-PCR assays on the TPX2 production in GC cells. **(D)** Outcomes from CCK-8 assays on the viabilities of cells MKN-45 and AGS. **(E, F)** Outcomes from transwell assays on the number of migrated GC cells. **(G, H)** Outcomes from transwell assays on the number of infiltrated GC cells. **p<0.01, ***p<0.001, ****p<0.0001.

To delve into the interference of TPX2 overproduction with GC cells’ malignant traits, OE-TPX2 was transfected into cells AGS and MKN-45, which were then tested following western blot and qRT-PCR procedures to make clear the TPX2 production. As substantiated by the outcomes, the TPX2 protein produced more post-transfection of OE-TPX2 (**Figures 2A, B**) and so did the TPX2 mRNA (**Figure 2C**). This outcome paved the way for subsequent functional assays. By CCK-8 assays, cell viability influenced by TPX2 overproduction was assessed. In consequence, the viability of cells AGS and MKN-45 was intensified post-overproduction of TPX2 (**Figure 2D**). By transwell assays on the interference of TPX2 overproduction with cells’ motility and infiltration capacity, cells AGS and MKN-45 were detected to migrate more (**Figures 2E, F**) and infiltrate more (**Figures 2G, H**) after TPX2 overproduction. Above all, overproducing TPX2 intensified the viability, as well as motility and infiltration capacities, of GC cells.

### 3.3 The interference of TPX2 attenuation with the biological activities of GC cells

**Figure 3:**
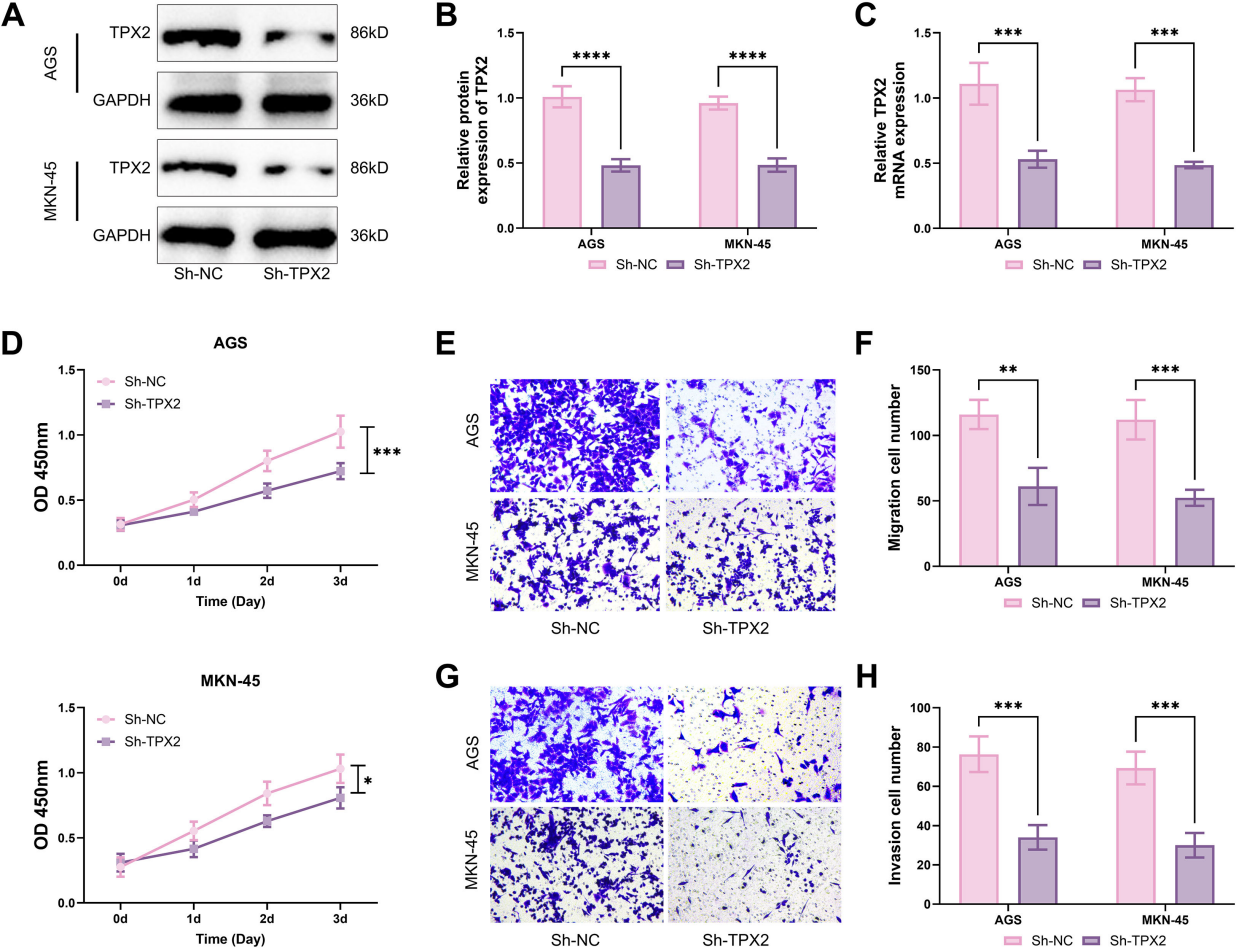
The interference of TPX2 attenuation with the biological activities of GC cells. **(A, B)** Sh-TPX2 was transfected into AGS and MKN-45 cells, and TPX2 silencing efficiency was assessed through western blot. **(C)** Outcomes from qRT-PCR assays on the TPX2 production in GC cells. **(D)** Outcomes from CCK-8 assays on the viabilities of cells MKN-45 and AGS. **(E, F)** Outcomes from transwell assays on the number of migrated GC cells. **(G, H)** Outcomes from transwell assays on the number of infiltrated GC cells. *p<0.05, **p<0.01, ***p<0.001, ****p<0.0001.

To make clear the biological effects of TPX2 downregulation in GC cells, Sh-TPX2 was transfected into cells AGS and MKN-45. Analysis through western blot and qRT-PCR confirmed a sharp drop in the production of both TPX2 protein and mRNA post-transfection (**Figures 3A-C**), thus enabling further functional testing. Over CCK-8 assays, cells AGS and MKN-45 were found markedly less active post-silencing of TPX2 (**Figure 3D**). Additionally, the numbers of migrated cells and infiltrated cells also fell off notably post-silencing of TPX2, as detected by transwell assays (**Figures 3E-H**). To sum up, TPX2 silencing retarded the viability, as well as motility and infiltration capability, of GC cells (**Figures 3E-H**), which is opposite to the outcomes of TPX2 overexpression.

### 3.4 TPX2’s modulation of the Wnt/β-catenin pathway in GC cells

**Figure 4:**
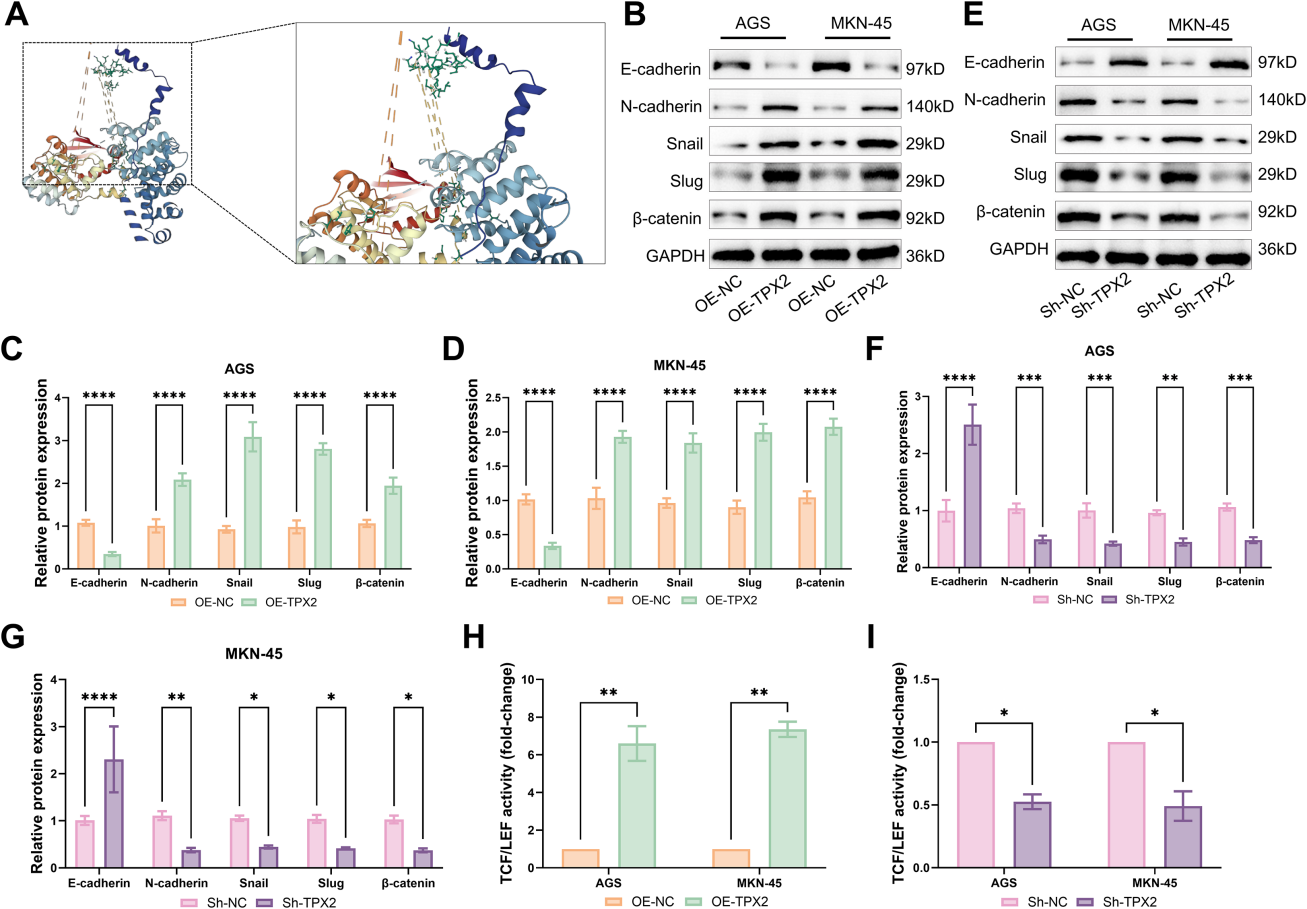
TPX2’s modulation of the Wnt/β-catenin pathway in GC cells. **(A)** Molecular docking results of TPX2 with β-catenin. **(B-D)** Outcomes from western blot assays on EMT-linked proteins levels post-overexpression of TPX2. **(E-G)** Outcomes from western blot assays on EMT-linked proteins levels post-silencing of TPX2. **(H, I)** Outcomes from Top-Flash assays on the activity of TCF/LEF. *p<0.05, **p<0.01, ***p<0.001, ****p<0.0001.

Molecular docking results showed that the Z-score of the optimal complex model combining the TPX2 and β-catenin was as high as 1203.548, which was much higher than the default binding confidence threshold of the ZDOCK platform, suggesting that the two can form a stable and direct binding conformation at the molecular level (**Figure 4A**). Subsequently, western blot procedures were followed to assess the production of proteins linked to EMT and proteins linked to the Wnt/β-catenin pathway. After TPX2 overexpression in AGS and MKN-45 cells, there was a large decrease in the E-cadherin protein production but an evident rise in the contents of β-catenin protein, N-cadherin, Snail, and Slug (**Figures 4B-D**). This suggested that TPX2 overexpression accelerated the EMT and enabled the Wnt/β-catenin pathway. In contrast, TPX2 underexpression stimulated a high elevation in the E-cadherin protein content, and the contents of β-catenin, N-cadherin, Snail, and Slug (**Figures 4F, G**). This denoted that TPX2 underexpression suppressed the EMT and blocked the Wnt/β-catenin pathway. TOP-Flash is a reporter plasmid employed for assessing the β-catenin-mediated TCF/LEF transcriptional activity within the Wnt pathway (28). Hence, TOP-Flash reporter assays were carried out further to affirm the interference of TPX2 with the Wnt/β-catenin axis. As an outcome, TPX2 overproduction strengthened the TCF/LEF transcriptional activity, and vice versa (**Figures 4H, I**). This substantiated the great role of TPX2 in enabling the Wnt/β-catenin pathway.

### 3.5 TPX2’s intervention in GC vessel formation

**Figure 5:**
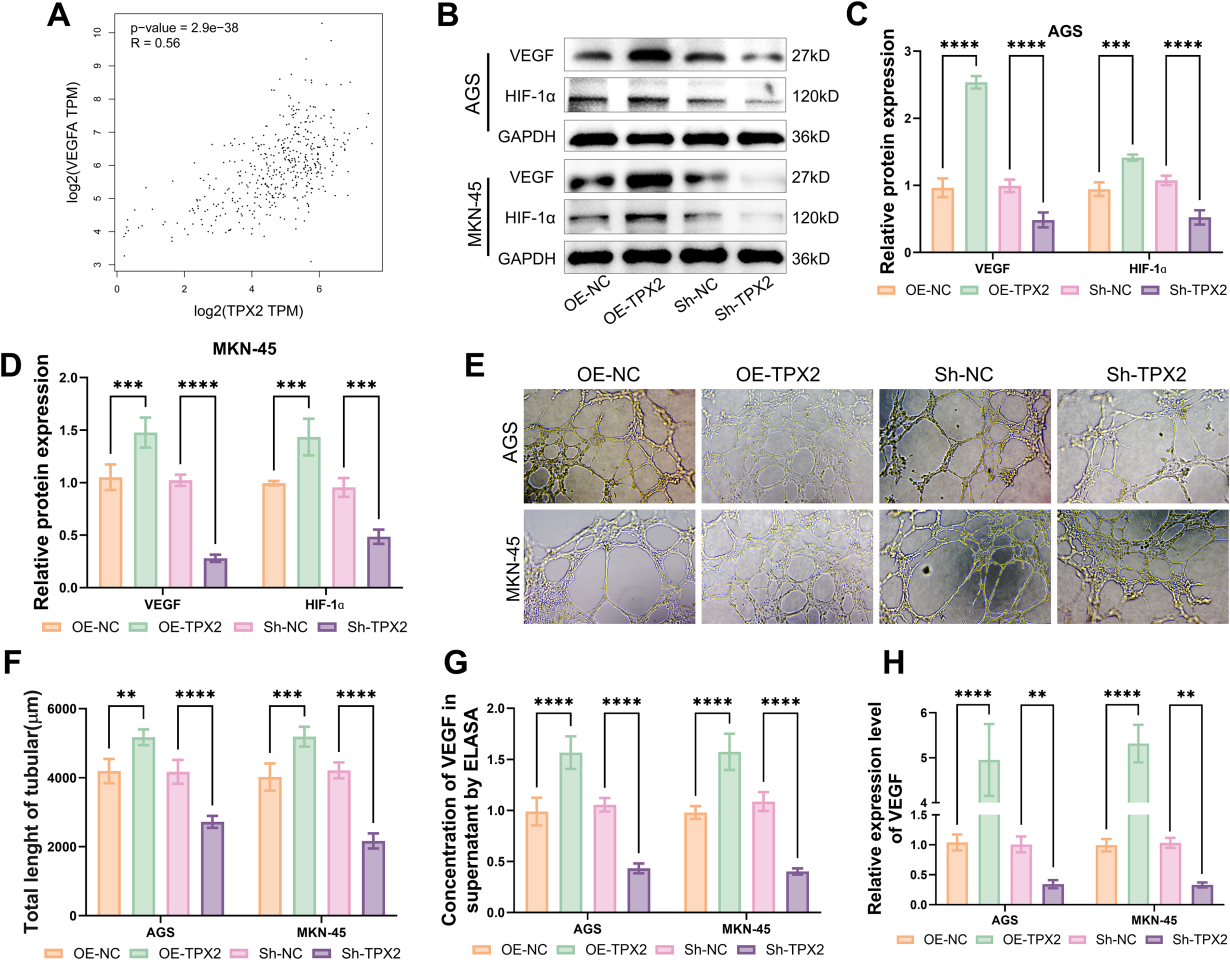
TPX2’s intervention in GC vessel formation. **(A)** TPX2 is positively correlated with VEGF as predicted by GEPIA. **(B-D)** Outcomes from western blot assays on the production of VEGF and HIF-1α. **(E, F)** Outcomes from vessel formation assays on the vessel formation ability of HUVECs. **(G)** Outcomes from ELISA on the VEGF content. **(H)** Outcomes from qRT-PCR assays on the VEGF mRNA production. **p<0.01, ***p<0.001, ****p<0.0001.

From the GEPIA database, TPX2 was found positively related to VEGF in production (**Figure 5A**). Western blot outcomes showed that overproducing TPX2 notably intensified the protein production of VEGF and HIF-1α in cells AGS and MKN-45, whereas attenuating TPX2 led to a sharp decline in that production (**Figures 5B-D**). The vessel formation assay provided more direct evidence that overproducing TPX2 sped up the GC angiogenesis while silencing TPX2 brought the opposite effect (**Figures 5E, F**). What’s more, ELISA and qRT-PCR outcomes unveiled that overproducing TPX2 greatly heightened the VEGF content in cells AGS and MKN-45 (**Figure 5G**) and so did the VEGF mRNA content (**Figure 5H**). In contrast, silencing TPX2 resulted in a great fall in both VEGF content and VEGF mRNA content. This confirmed that TPX2 can enhance GC vessel formation.

### 3.6 The efficacy of Wnt/β-catenin pathway suppression to partly weaken the GC aggravation promoted by TPX2

**Figure 6:**
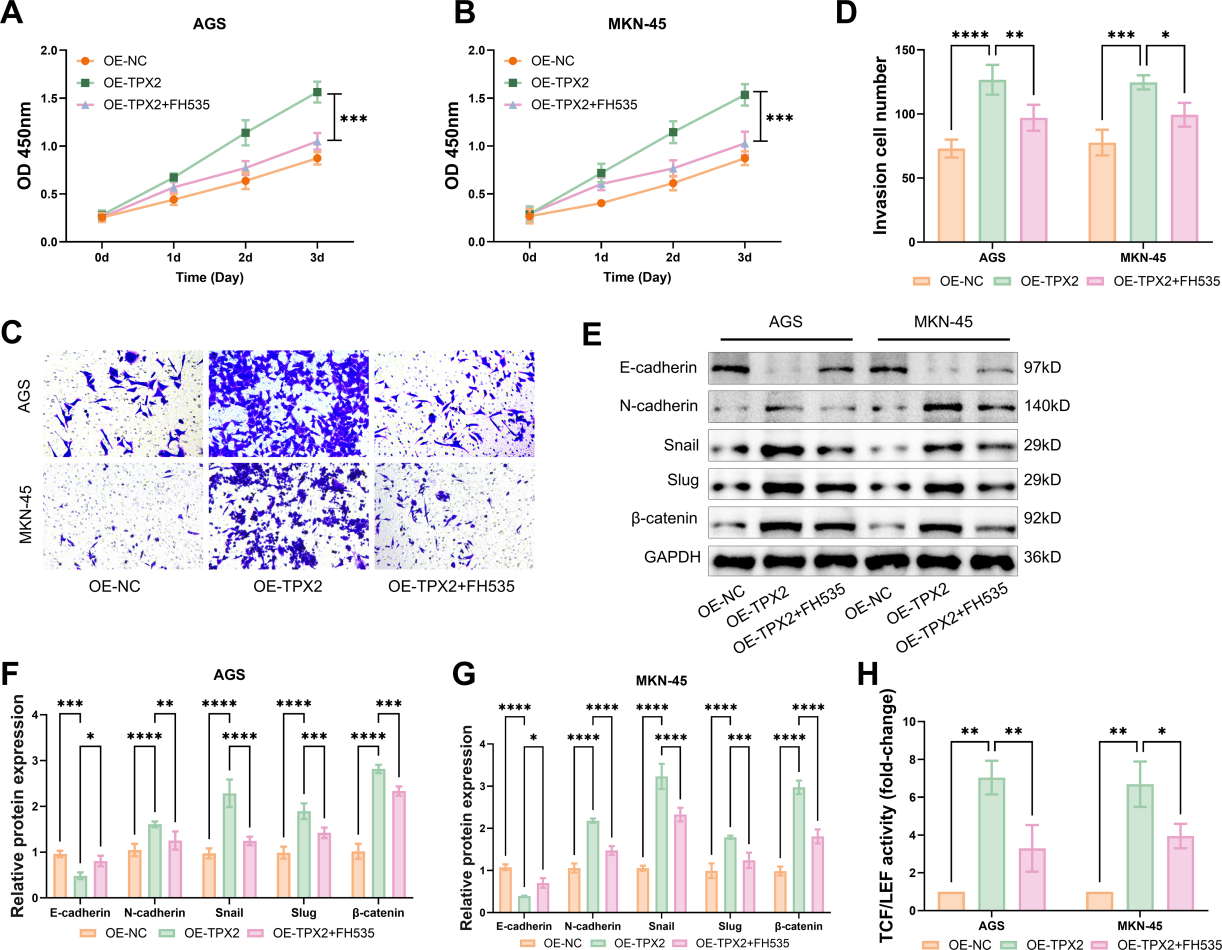
The efficacy of Wnt/β-catenin pathway suppression to partly weaken the GC aggravation promoted by TPX2. **(A, B)** Following intervention with the pathway inhibitor FH535, the viabilities of cells AGS and MKN-45 were assessed through CCK-8 assay. **(C, D)** Transwell assays of the number of infiltrated GC cells. **(E-G)** Western blot analysis of the production of EMT-linked proteins. **(H)** Outcomes from Top-Flash assays on the activity of TCF/LEF. *p<0.05, **p<0.01, ***p<0.001, ****p<0.0001.

To confirm if TPX2 drives the exacerbation of GC via the Wnt/β-catenin pathway, the interference of a pathway inhibitor FH535 (20 μmol/L) with the GC cell viability was evaluated through CCK-8 assays. Resultantly, cells AGS and MKN-45 became more viable after TPX2 overproduction; this phenomenon however was weakened somewhat with the administration of FH535 (**Figures 6A, B**). Further, transwell infiltration assays uncovered that these two cells’ infiltration driven by overproducing TPX2 could be attenuated partly by the FH535 administration (**Figures 6C, D**). Western blot results indicated that these two cells’ EMT facilitated by overproducing TPX2 and the production of β-catenin protein could be retarded somewhat by the FH535 administration (**Figures 6E-G**). In the TOP-Flash assay, FH535 notably offset the increase in TCF/LEF transcriptional activity induced by overexpressing TPX2 (**Figure 6H**). These consequences further proved that TPX2 facilitated GC cell worsening by enabling the Wnt/β-catenin pathway.

### 3.7 Downregulating TPX2 inhibits tumor growth via Wnt/β-catenin pathway

**Figure 7:**
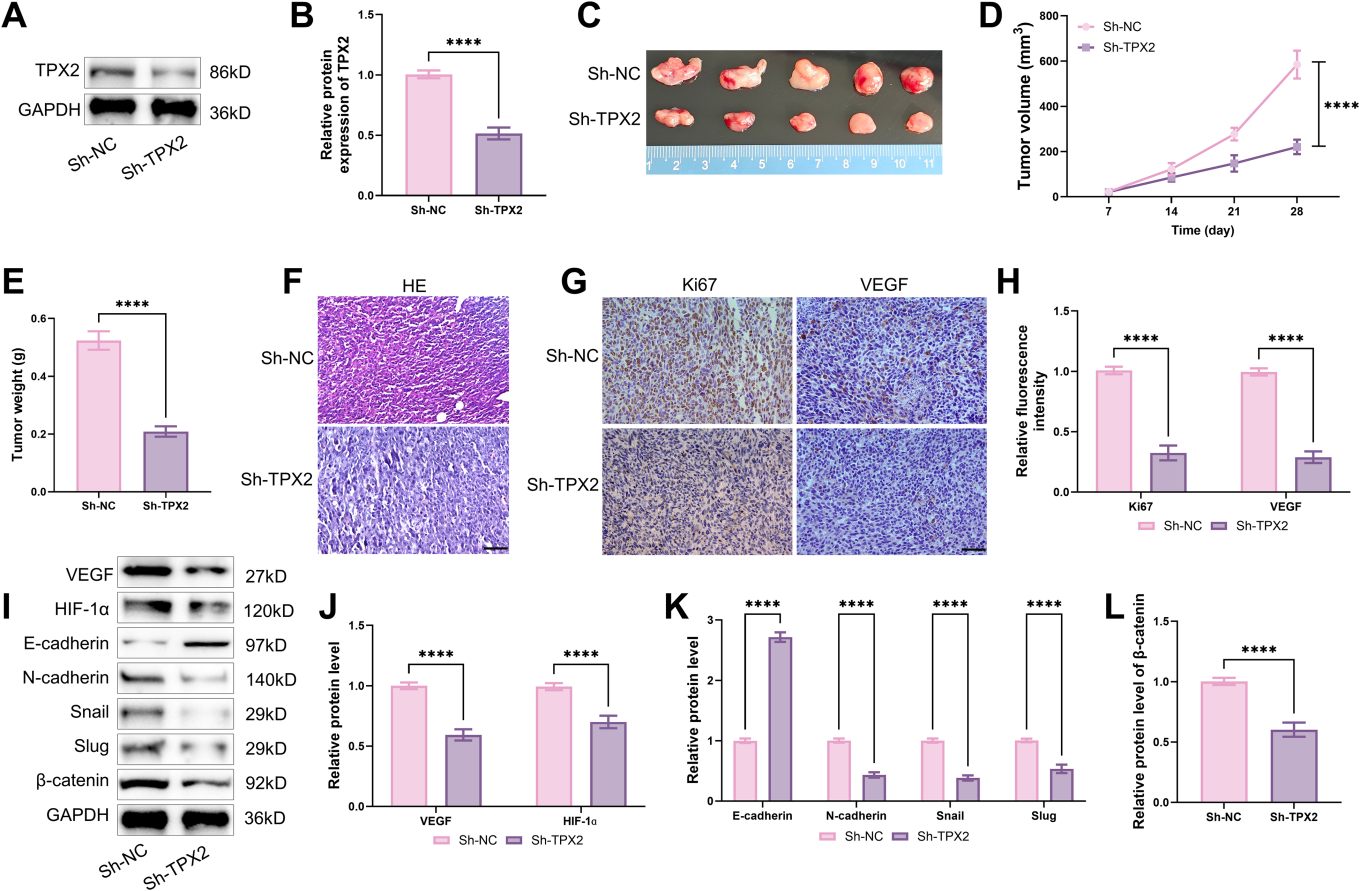
Downregulating TPX2 suppresses tumor growth by modulating Wnt/β-catenin pathway. **(A, B)** Western blot indicated that AGS cells transfected with Sh-TPX2 exhibited reduced TPX2 levels in tumor tissues. **(C)** On day 28, the tumors were excised from the mice and photographed for documentation. **(D)** The volume of subcutaneous tumors in mice was recorded weekly, and tumor growth curves were plotted. **(E)** On d28, tumors were excised and weighed. **(F)** HE staining revealed that TPX2 knockdown disrupts the structure of tumor tissue. **(G, H)** Immunohistochemical analysis revealed that TPX2 knockdown reduced CD31, Ki67 and VEGF levels in tumor tissues. (**I-L)** Western blot analysis of VEGF and HIF-1α, N-cadherin, Snail, Slug, β-catenin, and E-cadherin levels in tumor tissues following TPX2 knockdown. ****p<0.0001.

Finally, a GC mouse model was established to investigate whether TPX2 knockdown could also suppress tumor growth *in vivo*. Following injection and transfection of AGS cells with Sh-TPX2, TPX2 protein level was markedly downregulated in tumor tissues (**Figures 7A, B**), and tumor volume and weight were also notably reduced (**Figures 7C-E**). HE staining revealed higher cellular density in tumor tissues of the Sh-NC group. However, tumor tissues in the Sh-TPX2 group exhibited loose cellular arrangement and increased nuclear condensation, indicating that TPX2 knockdown effectively disrupts tumor tissue architecture and enhances cellular damage (**Figure 7F**). Immunohistochemical results demonstrated that knockdown TPX2 notably lowered the cell proliferation marker Ki67, endothelial cell marker CD31, and the key angiogenesis factor VEGF levels in tumor tissues (**Figure 7G, H**). Additionally, following TPX2 knockdown, VEGF, HIF-1α, Snail and Slug, and the stromal marker N-cadherin levels were notably reduced in tumor tissues. Conversely, E-cadherin expression was markedly elevated, while β-catenin expression was significantly suppressed (**Figures 7I-L**). The above experimental results collectively confirm that TPX2 knockdown effectively suppresses cell proliferation, angiogenesis, and tumor growth by regulating the Wnt/β-catenin pathway.

## 4 Discussion

**Figure 8:**
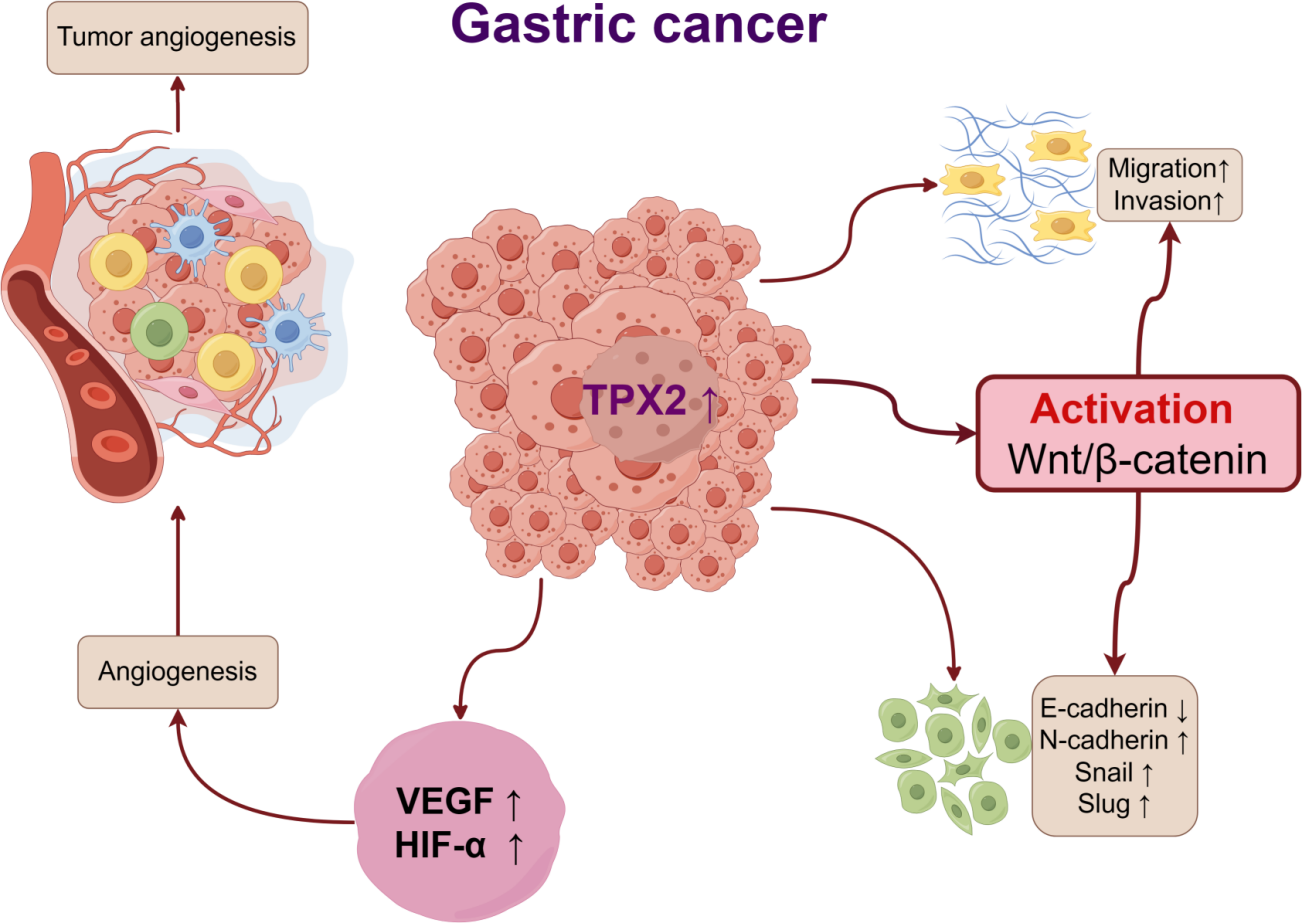
Diagram of TPX2’s promotion of the GC advancement by enabling the Wnt/β-catenin pathway.

The present work uncovered a notably high TPX2 production in GC tissues and cells; overexpression of TPX2 increased the expression of Wnt/β-catenin pathway-related proteins, resulting in a sharp decline in the cells’ E-cadherin protein production but a high rise in the N-cadherin, Snail, and Slug production, facilitating GC cells to migrate and infiltrate; consequently, VEGF and HIF-1α levels were elevated, contributing to the tumor vessel formation (**Figure 8**). Downregulating TPX2 inhibited the Wnt/β-catenin pathway, consequently hindering cell proliferation and angiogenesis, ultimately inhibiting tumor growth in nude mice.

TPX2, situated on chromosome 20q11.21, is essential for correct spindle formation and normal cell division (29, 30). Studies have repeatedly unraveled that downregulating the TPX2 level may be conducive to treating cancers. Specifically, Shaath et al. used bioinformatics to reveal that TPX2 production is elevated in colorectal cancer, and the *in-vivo* and *in-vitro* growth, motility, and infiltration of such cancer cells can be impeded by impairing the TPX2 production (31). Yang et al. surveyed and reported that both TPX2 protein and mRNA production were remarkably higher in breast cancer tissues; the expansion, motility, and infiltration of such cancer cells were suppressed post-downregulation of TPX2 (32). Furthermore, Liang et al. discovered that underexpressing TPX2 can lower the survival rate of GC cells, inducing apoptosis and ceasing the cell cycle at the G2/M stage (33). By the present survey, TPX2’s high production in GC was evidenced in databases GEPIA and TCGA, which was also the case in GC patients’ tissues and GC cell lines according to the qRT-PCR and western blotting consequences and accords with the study results of Shao et al. (34). Moreover, enhancing TPX2 production could enhance GC cells’ viability, motility, and infiltration, while silencing TPX2 offset this trend.

The swift proliferation of tumors relies heavily on an adequate blood supply, which underscores the importance of vessel formation for tumor advancement (35, 36). Tumor vessel formation is mediated by a complex interplay of cytokines that either support or inhibit vessel formation, along with several signaling pathways, including VEGF, HIF-1α, angiogenin, fibroblast growth factors, and transforming growth factor β (37, 38). By interacting with three important tyrosine kinase receptors (VEGFR1, VEGFR2, and VEGFR3), VEGF family members initiate a series of intracellular signal transduction pathways and stimulate endothelial cells to undergo mitosis and form capillaries, hence their critical contribution to vessel formation in tumors (39, 40). A critical transcriptional regulator HIF-1α can stimulate the transcription of angiogenesis-related genes and their receptors, including VEGF, ANGPT1, PlGF, and PDGFB, enhancing the progress of a cell cycle and DNA replication, thus its high significance for angiogenesis (41, 42). In cancers, HIF-1α production is heightened and supports tumor advancement or infiltration by accelerating vessel formation and altering metabolic processes (43). In our work, GC cells exhibited higher VEGF and HIF-1α contents following the TPX2 overproduction while the opposite outcome was detected following the TPX2 downregulation. This demonstrated the contribution of TPX2 to GC vessel formation.

GC onsets and progresses intricately, involving the regulation of multiple signaling pathways (44, 45). Shan et al. unraveled that SERPINH1 mediates EMT and GC advancement via the Wnt/β-catenin axis (46). Ding et al. concluded that silencing TPX2 deactivates the Wnt/β-catenin pathway and mediates proteins linked to cell cycles and apoptosis, thus preventing liver tumor cells from proliferating and inducing apoptosis (47). Our work revealed that overexpression of TPX2 increased the activity of the Wnt/β-catenin pathway, enhancing the EMT in GC cells. Results from the TOP-Flash reporter gene assay further substantiated that TPX2 enables this pathway. Interestingly, Wnt/β-catenin pathway inhibitor FH535 attenuated the promotion of STAD malignant progression by overexpression of TPX2, suggesting that TPX2 may promote STAD malignant progression through activation of the Wnt/β-catenin pathway.

This study still has limitations. Future research could include immunoprecipitation, immunofluorescence co-localization, and nuclear-cytoplasmic separation experiments to further verify the endogenous direct interaction between TPX2 and β-catenin. Furthermore, future studies could further confirm the causal relationship between TPX2 and β-catenin at the functional level by knocking down β-catenin, transfecting dominant-negative TCF/LEF constructs, or overexpressing β-catenin in TPX2-silenced cells.

## 5 Conclusion

In summary, TPX2 was highly expressed in STAD tissues and cells, and overexpression of TPX2 increased the expression of proteins related to the Wnt/β-catenin pathway, which in turn increased the viability of STAD cells and promoted migration, invasion and tumor angiogenesis. This research elucidates the mechanism by which TPX2 promotes malignant development in GC, providing experimental evidence and theoretical reference for TPX2 as a viable treatment target for GC. However, our study focuses on the Wnt/β-catenin pathway. Future research may explore whether TPX2 participates in the malignant progression of GC through synergistic regulation of other signaling pathways (including MAPK, PI3K/Akt, etc.). The complexity of its regulatory network requires further elucidation.

## Data Availability

The original contributions presented in the study are included in the article/supplementary material. Further inquiries can be directed to the corresponding author.
